# Correlation of Airway POCUS Measures with Screening and Severity Evaluation Tools in Obstructive Sleep Apnea: An Exploratory Study

**DOI:** 10.3390/jcm14144858

**Published:** 2025-07-09

**Authors:** Sapna Ravindranath, Yatish S. Ranganath, Ethan Lemke, Matthew B Behrens, Anil A. Marian, Hari Kalagara, Nada Sadek, Melinda S. Seering, Linder Wendt, Patrick Ten Eyck, Rakesh V. Sondekoppam

**Affiliations:** 1 Department of Anesthesia, Indiana University School of Medicine, Indianapolis, IN 46202, USA; drsapna.r@gmail.com (S.R.); yrangan@iu.edu (Y.S.R.); 2 Department of Emergency Medicine, University of Michigan Health-West, Wyoming, MI 49519, USA; ethan.lemke22@gmail.com; 3 Kent Hospital, Warwick, RI 02886, USA; matthew.b.behrens@gmail.com; 4 Department of Anesthesia, University of Iowa Carver College of Medicine, Iowa City, IA 52242, USA; anil-marian@uiowa.edu (A.A.M.); nada-sadek@uiowa.edu (N.S.); melinda-seering@uiowa.edu (M.S.S.); 5 Department of Anesthesia, Mayo Clinic, Jacksonville, Fl 32224, USA; drkalagara@gmail.com; 6 Institute for Clinical and Translational Science, University of Iowa, Iowa City, IA 52242, USA; linder-wendt@uiowa.edu (L.W.); patrick-teneyck@uiowa.edu (P.T.E.); 7 Department of Anesthesia, Pain and Perioperative Medicine, Stanford University, Palo Alto, CA 94304, USA

**Keywords:** obstructive sleep apnea, point of care ultrasound, perioperative medicine

## Abstract

**Background:** Obstructive Sleep Apnea (OSA) is a common occurrence in the perioperative patient population but is often undiagnosed. Point-of-Care Ultrasound (POCUS) has emerged as a promising tool for perioperative assessment; however, its effectiveness in detecting the presence or severity of OSA needs to be evaluated. **Objective:** We assessed the ability of airway POCUS as a screening and severity evaluation tool for OSA by examining its correlation with STOP-BANG scores and the Apnea–Hypopnea Index (AHI). **Design:** Cross-sectional observational study. **Setting:** A single-center study in a tertiary care hospital between June 2020 to May 2021. **Patients:** Adult patients aged 18–65 with prior Polysomnography (PSG) for OSA workup were screened. **Interventions:** The participants completed the STOP-BANG questionnaire and subsequently underwent POCUS examinations, either pre- or post-surgery. Ten different POCUS views previously used for evaluating OSA were acquired in a predefined sequence, with subsequent measurements of airway parameters. **Outcome measures:** Generalized linear modeling was used to explore and assess the relationships between the measured parameters, STOP-BANG, and AHI scores (modeled continuously and categorized into risk levels of STOP-BANG and AHI). **Results:** A total of 260 patients were screened, of which 142 were enrolled and 127 completed the scanning studies. The median AHI was 16.71, while the STOP-BANG scores were mostly between 5 and 6, indicating a moderate-to-high OSA risk in the study population. Notably, only neck circumference was significantly associated with AHI severity (*p* = 0.012), whereas none of the other POCUS measures were. Among the POCUS measures, significant associations with STOP-BANG scores were observed for the Tongue Cross-Sectional Area (T-CSA) (*p* = 0.002), Retro-Palatal Diameter (RPD) (*p* = 0.034), Distance Between Lingual Arteries (DLA) (*p* = 0.034), and Geniohyoid Muscle Thickness (GMT) (*p* = 0.040). **Conclusions:** Neck circumference is a more reliable predictor of OSA severity (AHI) compared to other POCUS measurements. Many of the POCUS measures had a good correlation with the STOP-BANG scores, highlighting the utility of POCUS as a screening tool for OSA rather than as a severity evaluation tool.

## 1. Introduction

Obstructive Sleep Apnea (OSA) is recognized as a significant concern in perioperative care because of its impact on postoperative recovery profiles and association with postoperative cardiovascular and respiratory morbidity [1,2,3]. The prevalence of OSA is notably high and continues to rise [4,5], establishing it as a common comorbidity encountered by anesthesiologists in current practice. Despite its known prevalence, a significant proportion of patients presenting for anesthesia and surgery remain undiagnosed with OSA [6,7]. Beyond respiratory issues, OSA patients may also suffer from cardiovascular problems such as hypertension, ischemic heart disease and cardiac failure, pulmonary hypertension, cerebrovascular disease, metabolic syndrome, and depression, among other comorbidities [8,9,10]. Over the past decade, a growing body of evidence has highlighted OSA as an independent risk factor for numerous complications [1,11]. The perioperative phase is a vulnerable period for these patients as the effects of general anesthesia and narcotics may exacerbate the underlying comorbidities associated with OSA or lead to adverse events [2,3,12].

Anesthesia societies have emphasized the importance of the pre-surgical identification of patients with moderate-to-severe OSA as a strategy to prevent both major and minor perioperative complications [13,14]. Questionnaire-based screening tools, such as the perioperative-friendly STOP-BANG questionnaire, Berlin Questionnaire, or Epworth Sleepiness Scale on subjective scoring components, are often employed for the identification of OSA. While these tests are sensitive, they often lack specificity, resulting in many false positives [15,16,17]. Additionally, questionnaire-based screening is not feasible in many patient populations who may have difficulty understanding the test or are unaware of the answers to key questions such as “observed apnea.” Polysomnography (PSG), the gold standard for diagnosis, involves the overnight monitoring of activities during sleep such as brain waves, pulse oximetry, heart rate, breathing, and eye and leg movements. Despite its diagnostic accuracy, PSG is expensive and less accessible, which underscores the need for refined screening methods [18].

Given these challenges, Perioperative Point-Of-Care Ultrasound (POCUS), which is being explored beyond its established uses for diagnosing OSA [19,20], might offer an alternative screening and evaluation solution for OSA. Early research shows promising correlations between POCUS measurements and OSA severity [21,22,23], suggesting that POCUS might not only provide an objective method by which to address specific issues associated with traditional questionnaires but might also serve as a viable screening tool when questionnaires are impractical. While OSA is a dynamic process, with the narrowing of the airways during sleep, it is well known that resting state measurements in awake patients have shown the phenotypic characteristics of a narrower upper airway and a propensity for increased collapsibility, with diminishing muscle tone during sleep [23,24,25,26,27]. Hence, various static and dynamic POCUS measures have been used to assess the presence and severity of OSA, but not all of these measures have been used in a single cohort of patients.

With this background, we aimed to explore the potential of POCUS as a screening and severity evaluation tool in patients with OSA by investigating the correlation between specific POCUS measures and STOP-Bang scoring, as well as the Apnea–Hypopnea Index (AHI). We hypothesized that specific POCUS parameters, including the Distance between Lingual Arteries (DLA), Geniohyoid Muscle Thickness (GMT), Lateral Parapharyngeal Wall Thickness (LPWT), Tongue Base Thickness (TBT), and Cross-Sectional Area of the Tongue in the Sagittal plane (CSAT-S), can predict the presence and severity of OSA. We further anticipated that these parameters would correlate positively with both the AHI and STOP-Bang scores.

## 2. Methods

**Ethics:** The study received approval from the University’s Institutional Review Board (HAWK IRB ID 202001068, approved on 30 April 2020; IRB chair: Dr. Catherine Woodman) and was registered at Clinicaltrials.gov (NCT04443582, Principal Investigator: Rakesh Sondekoppam; date of registration: 23 June 2020).

### 2.1. Study Design and Setting

This cross-sectional observational study was conducted over a one-year period at The University of Iowa Healthcare from June 2020 to May 2021. Eligible participants were those scheduled to undergo elective surgery at the University of Iowa Hospitals and Clinics, identified through chart reviews in either the Pre-anesthesia Evaluation Clinic or the Pre-operative Holding Area.

### 2.2. Study Population

The inclusion criteria were patients of any sex, aged 18–65 years, with a patient-reported history of PSG-diagnosed Obstructive Sleep Apnea (OSA) within the last 4 years. Exclusion criteria included those without a PSG performed within the last 4 years, individuals with central sleep apnea, patients who had undergone or were scheduled for surgeries related to craniofacial or oropharyngeal abnormalities, recent oropharyngeal surgery post-PSG, ENT tumors, history of ENT/neck surgeries, those aged < 18 or >65 years, inability to provide consent, and non-English speakers.

### 2.3. Recruitment and Scanning Technique

Potential participants were briefed on the objectives and methods of the study. Once informed consent was obtained, the participants were assigned a unique study number. Before scanning, the participants completed the STOP-BANG Questionnaire (SBQ) as part of their standard pre-surgical evaluation, and neck circumference measurements were taken if not already available from the STOP-BANG questionnaire.

A member of the research team, blinded to the STOP-BANG scores and polysomnography results, performed Point-Of-Care Ultrasonography (POCUS). All POCUS practitioners had the requisite experience, having conducted at least 20 patient scans prior to participating in the study, ensuring a standardized scanning technique and accurate identification of anatomical landmarks.

Scanning was conducted either pre- or post-surgery depending on the type of surgery and its prerequisites. During scanning, the patients were positioned reclined, with the hyoid-external acoustic meatus axis nearly parallel to the ground, mouths closed, and were instructed to remain relaxed. Ten standard images were acquired in a predefined sequence: Tongue Base Thickness—Sagittal (TBT-S), Tongue Cross-Sectional Area (T-CSA), Upper Airway Length (UAL) (1); Tongue Base Thickness–Sagittal during Müller Maneuver (TBT-S MM), Upper Airway Length during Müller maneuver (UAL MM) (2); Skin–Hyoid Distance (SHD) (3); Retro-Glossal Diameter (RGD) (4); Transverse Diameter of Pharynx (TDP) (5); Retro-Palatal Diameter (RPD) (6); Tongue Base Thickness—Coronal (TBT-C), Geniohyoid Muscle Thickness (GMT) (7); Distance Between Lingual Arteries (DLA) (8); Right Lateral Pharyngeal Wall Thickness (R LPWT) (9); and Left Lateral Pharyngeal Wall Thickness (L LPWT) (10) (Supplementary A).

After scanning, participant engagement in this study was concluded, and no long-term follow-up was required. All data, including images, were securely archived. Each scan involved capturing images of specific parameters, allowing up to three images per view, if the sonographer determined the need for improved visualization. Optimal visualization was achieved by adjusting the depth and occasionally using Color Doppler imaging for artery identification. Images were saved under the participant’s study number and later transferred to a secure drive for measurements and interpretations by evaluators blinded to the clinical data using the RadiAnt DICOM viewer [28].

Given the exploratory nature of the study, our primary aim was to examine correlations between POCUS measures and STOP-BANG scores, as well as between POCUS measures and the Apnea–Hypopnea Index (AHI), both as ordinal data and continuous measures. For comparison, STOP-BANG scores were classified as low-risk (0–2), intermediate risk (3–4), and high risk (>4); whereas the AHI was categorized based on the absence (AHI < 5) or presence (AHI > 5) of obstructive sleep apnea. Additionally, POCUS measures were compared to AHI based on the severity of OSA, classified according to either the presence or absence of moderate-to-severe OSA (AHI > 15) or the presence or absence of severe OSA (AHI > 30).

### 2.4. Statistical Analyses

Data were summarized using medians and interquartile ranges for continuous variables and counts and percentages for categorical variables. Univariate generalized linear models assessed the relationships between outcomes, STOP-BANG scores, and AHI (both continuously and as categorized previously), along with various subject characteristic predictors. Outcome distributions were evaluated to ensure the appropriate specification of link functions, including logit for dichotomous outcomes, cumulative logit for ordinal outcomes, log for STOP-BANG scores (negative binomial distribution), and continuous AHI outcomes (gamma distribution). Mean or odds ratios were reported, where applicable, with 95% confidence intervals and *p*-values.

Receiver Operating Characteristic (ROC) curves were constructed to evaluate the relationship between neck circumference and severe AHI. This analysis determined the optimal threshold for dichotomizing neck circumference along with the estimated sensitivity and specificity. The Area Under the Curve (AUC) is a measure of the predictive strength of neck circumference in relation to severe AHI. All analyses were performed using R version 4.3.3. Statistical significance was set at *p* < 0.05.

## 3. Results

Between June 2020 and May 2021, 260 patients were screened for eligibility, of whom 142 provided written informed consent and were enrolled in the study. Of these, 127 underwent successful scanning, while 15 could not be scanned because of logistical issues. Sleep study data were unavailable for 41 of these 127 patients; however, all enrolled patients (n = 127) completed the STOP-BANG questionnaire before their hospital visit. Patient demographics and characteristics are presented in Table 1. The Apnea–Hypopnea Index (AHI) had a median value of 16 (IQR 8, 31). The distribution of STOP-BANG scores revealed a moderate-to-high risk of OSA, with scores of 5 and 6 being the most prevalent at 24% and 26%, respectively.

Table 2 presents various POCUS airway measurements and neck circumferences, reported as median and interquartile range, along with the number of missing data. Key ultrasound measurements include TBT-S at 6.8 cm (6.3–7.5); DLA at 3.0 cm (2.6–3.3); LPWT averaged 1.2 cm on the left and 1.1 cm on the right (1.0–1.4); RPD at 4.3 cm (3.6–4.7); UAL during Muller Maneuver at 2.5 cm (2.1–3.2); and neck circumference at 46 cm (42–50).

Tongue Cross-Sectional Area (T-CSA) was positively associated with STOP-BANG scores, with a Mean Ratio (MR) of 1.02 (95% CI: 1.00–1.05; *p* = 0.020) (Supplementary B: Table S1). Although statistically significant, the clinical relevance of this finding is nuanced owing to the modest effect size, suggesting that the impact in clinical settings may be limited. Further analyses using a cumulative logistic regression model explored the relationship between ultrasound measurements and categorization of STOP-BANG scores into high- (5–8/8), medium- (3–4/8), and low-risk (0–2/8) groups for Obstructive Sleep Apnea (OSA) (Supplementary B: Table S2). Tongue Base Thickness—Sagittal (TBT-S) was negatively associated with OSA severity (OR = 0.97; 95% CI: 0.94–1.00; *p* = 0.028), while Tongue Cross-Sectional Area (T-CSA) (OR = 1.21; 95% CI: 1.08–1.38; *p* = 0.002), Distance between Lingual Arteries (DLA) (OR = 2.55; 95% CI: 1.10–6.27; *p* = 0.034), Retro-Palatal Diameter (RPD) (OR = 1.69; 95% CI: 1.05–2.80; *p* = 0.034), and Geniohyoid Muscle Thickness (GMT) (OR = 6.07; 95% CI: 1.23–38.2; *p* = 0.040) all exhibited significant positive associations with STOP-BANG score categories.

Analyses of various ultrasonographic measurements, neck circumference, and Apnea–Hypopnea Index (AHI) (Supplementary B: Table S3) revealed that only neck circumference displayed a statistically significant relationship with AHI (MR = 1.05, 95% CI: 1.01–1.09; *p* = 0.012), indicating its potential as a reliable predictor of sleep apnea severity. Further analysis using a cumulative logistic model investigated the relationship between ultrasonographic measurements, neck circumference, and the ordinal AHI of none (AHI < 5 events/h), mild (AHI: 5–15 events/h), moderate (AHI: 15–30 events/h), and severe (AHI > 30 events/h) (Supplementary B: Table S4). These analyses found that neck circumference was the sole measurement significantly associated with ordinal AHI (OR = 1.09; 95% CI: 1.01–1.19; *p* = 0.037).

The association between various ultrasonographic measurements, neck circumference, and differing degrees of Apnea–Hypopnea Index (AHI) was investigated (Supplementary B: Tables S5–S7). We specifically analyzed the odds of having an AHI greater than 5, 15, and 30, which are indicative of the presence of sleep apnea, moderate sleep apnea, and severe sleep apnea, respectively. Among the ultrasound measurements considered, only the transverse diameter of the pharynx was significantly associated with the presence of sleep apnea (AHI > 5), suggesting that individuals with a wider pharynx might have an increased chance of sleep apnea (OR = 2.47, 95% CI: 1.12–6.32; *p* = 0.037) (Supplementary B: Table S5). However, when exploring AHIs greater than 15 and 30 (Supplementary B: Tables S6 and S7), none of the measurements were significantly associated with moderate or severe sleep apnea.

Receiver Operating Characteristic (ROC) analysis for diagnosing moderate-to-severe AHI found that a neck circumference threshold of 43.4 cm could diagnose these conditions with a specificity of 0.613 and a sensitivity of 0.694, achieving an Area Under the Curve (AUC) of 0.637 (Supplementary B: Figure S1). Lastly, a strong association was observed between STOP-BANG scores greater than 4 and the presence of moderate-to-severe AHI, reinforcing previous evidence from the study population. This relationship was quantitatively assessed, indicating that individuals with a STOP-BANG score > 4 were nearly seven times more likely to exhibit moderate or severe sleep apnea (OR = 6.96, *p* = 0.0004) (Supplementary B: Table S8).

## 4. Discussion

Our study explored the relationship between sonographically measured airway anatomical characteristics (POCUS measurements) with STOP-BANG scores and the POCUS measurements or neck circumference with sleep apnea severity as gauged by the Apnea–Hypopnea Index (AHI). Our approach offers new insights into the anatomical markers of OSA. While there were significant associations between specific anatomical measurements (T-CSA, RPD, DLA, and GMT) and STOP-BANG score categories (Supplementary B: Table S2), only neck circumference was significantly correlated with AHI severity. Most other measurements showed no strong association with the AHI. The cross-sectional area of the tongue was significantly associated with the STOP-BANG score, although its clinical impact appears limited due to the small effect size. It is possible that neck circumference is a simpler and a more reliable marker for the presence of OSA than other measures. While we did not explore the predictive ability of a combination of POCUS measures, future studies should explore if a combination of some or all of the measures in question, such as T-CSA, RPD, DLA, and GMT, may have better predictability in detecting OSA or its severity.

A notable strength of our study is the inclusion of multiple POCUS measurements for OSA in the perioperative setting, unlike most studies that focus on singular measurements in non-surgical populations. This broader approach offers a more holistic understanding of how different anatomical markers interact with and contribute to OSA. Furthermore, our dual-analysis approach, examining anatomical measures in relation to both the Apnea–Hypopnea Index (AHI) and the STOP-BANG questionnaire, enhances the depth and applicability of our findings. It bridges the gap between isolated anatomical findings and their collective implications in OSA presentations while also highlighting the correlations between anatomical markers, OSA severity, and potential risk factors.

The divergence of our study from previous findings, in which POCUS airway measures, such as the distance between lingual arteries (>30 mm), resting tongue thickness (>60 mm), lateral pharyngeal wall thickness, and retro-palatal diameter shortening di, had moderate-to-strong correlations with OSA [29,30,31,32,33], needs discussion. Perhaps the inclusion of non-OSA patients may have led to better discrimination of the POCUS measures from those harboring OSA patients but recruiting patients with a sleep study and a negative diagnosis for OSA was found to be challenging before we began the study. The composition of our study cohort, predominantly consisting of patients diagnosed with OSA, has the issues inherent to observational study design since we wanted to perform measurements in patients with a known diagnosis of OSA who had a PSG within the past 4 years. While our study lacked a control group of healthy non-OSA patients and was exploratory in nature, the anatomical measurements of narrower airway phenotypes with a propensity for collapse have long been well known—even before the advent of POCUS measurements [25,26,27]. While our study had a good correlation of certain POCUS measures with STOP-BANG scores, a poor correlation was seen with AHI. Given the multifaceted nature of OSA encompassing both anatomical and physiological components, sole reliance on POCUS measurements might perhaps only identify the presence of OSA rather than the severity of it.

Nevertheless, our study has inherent limitations. While we did find a correlation between POCUS measures and STOP-BANG scores, the observational nature of this study was necessary in order to test our hypothesis, and we realize that the lack of non-OSA controls may limit the generalizability of our findings. Moreover, while static airway measurements have been well documented to indicate the presence of OSA and airway phenotypes with a propensity for collapse, it remains to be seen if our findings diverge when measurements are performed under sleep conditions. Despite procedural standardization and adequate expertise before scanning, ultrasound techniques have limitations in terms of examiner subjectivity. Furthermore, we did not validate our findings using advanced imaging techniques such as Magnetic Resonance Imaging (MRI) or Computed Tomography (CT).

In conclusion, among the various measurements for OSA screening, we found associations between specific POCUS airway measures and STOP-BANG scores. POCUS measures did not correlate with the severity of OSA, as determined by AHI, but neck circumference did correlate. The clinical utility of POCUS as a screening tool for OSA needs confirmation, especially in diverse patient populations. Further, it remains to be seen whether a combination of POCUS measures may have better discrimination on the presence and severity of OSA in surgical populations. Future studies should explore whether additional POCUS measures or combinations of measures could improve the accuracy of severity evaluation in OSA patients. Additionally, considering the multifaceted nature of OSA, incorporating physiological measures alongside anatomical POCUS measures may provide a more comprehensive evaluation of OSA severity and inform targeted interventions. The use of neck circumference as a determiner of harboring greater severity of OSA seems simpler but requires further evidence.

## Figures and Tables

**Table 1:** Patient characteristics and STOP-BANG scores.

Characteristic	Median (IQR)
Age	63.00 (23.00)
Height	168.90 (16.3)
Weight	99.70 (21.0)
BMI	34.48 (8.64)
**STOP-BANG score**	**Number of patients (percentage)**
0	1 (0.8%)
1	2 (1.6%)
2	7 (5.6%)
3	7 (5.6%)
4	20 (16%)
5	30 (24%)
6	33 (26%)
7	19 (15%)
8	7 (5.6%)

**Table 2:** POCUS airway measurements, Neck circumference and AHI.

Characteristic	Median (IQR)
Tongue Base Thickness—Sagittal (TBT-S) (cm)	6.80 (6.32, 7.49)
TBT-S during Muller Maneuver (cm)	6.78 (6.37, 7.43)
Tongue Base Thickness—Coronal (TBT-C) (cm)	6.67 (6.25, 7.31)
Upper Airway Length (UAL) (cm)	2.78 (2.20, 3.74)
UAL during Muller Maneuver (cm)	2.53 (2.12, 3.19)
Left Lateral Pharyngeal Wall Thickness (cm)	1.17 (0.95, 1.35)
Right Lateral Pharyngeal Wall Thickness (cm)	1.14 (0.93, 1.35)
Tongue Cross Sectional Area (T-CSA) (cm^2^)	24.8 (22.5, 27.1)
Distance between Lingual Arteries (DLA) (cm)	2.96 (2.63, 3.30)
Retro-Palatal Diameter (RPD) (cm)	4.26 (3.63, 4.69)
Retro-Glossal Diameter (RGD) (cm)	3.48 (2.71, 3.93)
Transverse Diameter of the Pharynx (cm)	3.86 (3.25, 4.38)
Geniohyoid Muscle Thickness (GMT) (cm)	0.83 (0.72, 1.00)
Skin-Hyoid Distance (SHD) (cm)	1.35 (1.07, 1.69)
Neck circumference (cm)	46 (42, 50)
AHI	16 (8, 31)

## Data Availability

Study data is available on request with the principal investigator.

## Supplementary Materials

The following supporting information can be downloaded at https://www.mdpi.com/article/10.3390/jcm14144858/s1.

## Abbreviations

AHI	Apnea–Hypopnea Index
BMI	Body Mass Index
CI	Confidence Interval
CRF	Case Report Form
CSAT-S	Cross-Sectional Area of the Tongue in the Sagittal Plane
DLA	Distance Between Lingual Arteries
ENT	Ear, Nose, and Throat
FDA	Food and Drug Administration
GMT	Geniohyoid Muscle Thickness
IQR	Interquartile Range
IRB	Institutional Review Board
LPWT	Lateral Parapharyngeal Wall Thickness
R LPWT	Right Lateral Parapharyngeal Wall Thickness
L LPWT	Left Lateral Parapharyngeal Wall Thickness
MRI	Magnetic Resonance Imaging
NCT	National Clinical Trial
OSA	Obstructive Sleep Apnea
POCUS	Point-of-Care Ultrasound
PSG	Polysomnography
RPD	Retro-palatal Diameter
RGD	Retro-glossal Distance
SBQ	STOP-BANG Questionnaire
SD	Standard Deviation
STOP-BANG	A screening tool for Obstructive Sleep Apnea (OSA) with eight components: “S” stands for significant Snoring; “T” for excessive Tiredness; “O” for Observed breathing cessations during sleep; “P” for high blood Pressure; “B” for BMI over 35 kg/m^2^; “A” for Age over 50 years; “N” for Neck circumference greater than 40 cm; and “G” for male Gender. Each element helps to assess OSA risk factors.
SHD	Skin–Hyoid Distance
TBT	Tongue Base Thickness
TBT-S	Tongue Base Thickness—Sagittal
TBT-C	Tongue Base Thickness—Coronal
T-CSA	Tongue Cross-Sectional Area
TDP	Transverse Diameter of Pharynx
UAL	Upper Airway Length
