# Supplementary material for: Correlation of Airway POCUS Measures with Screening and Severity Evaluation Tools in Obstructive Sleep Apnea: An Exploratory Study
Source: J Clin Med. 2025 Jul 9;14(14):4858. doi: 10.3390/jcm14144858 (PMC12294979; doi:10.3390/jcm14144858)
Supplement: Supplementary file 1 [file jcm-14-04858-s001.zip › jcm-3677877-supplementary.pdf]

Supplementary A:

A technical description of Point-of-Care Ultrasound imaging used in Obstructive Sleep Apnea

Technical description:

For patient positioning, a prone or reclined position with a pillow rolled behind the neck is useful to line up the orbitomeatal line perpendicular to the floor. The first step in using POCUS for OSA is to identify the tongue. Using a curvilinear probe, place the probe in the submental region in the mid-saggital plane. The hyperechoic, air-mucosa interface should be easily seen – though US depth settings may need to be changed. Once the tongue is visualized, slight lateral fanning of the probe can ensure you are getting the best view. Acquiring an image in this orientation allows you to measure the tongue base thickness in the sagittal plane (TBT-S), tongue cross sectional area (T-CSA), and upper airway length (UAL). TBT-S is measured from the skin to the hyperechoic line at the thickest part of the tongue. T-CSA here making sure to exclude geniohyoid. UAL is measured from the junction between geniohyoid and the hyoid bone, to the hyperechoic line (tongue) that is closest to it.

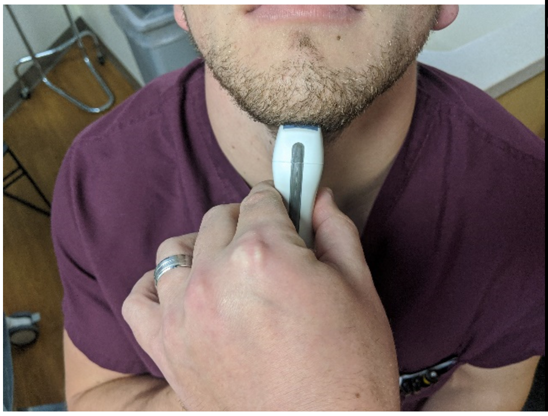

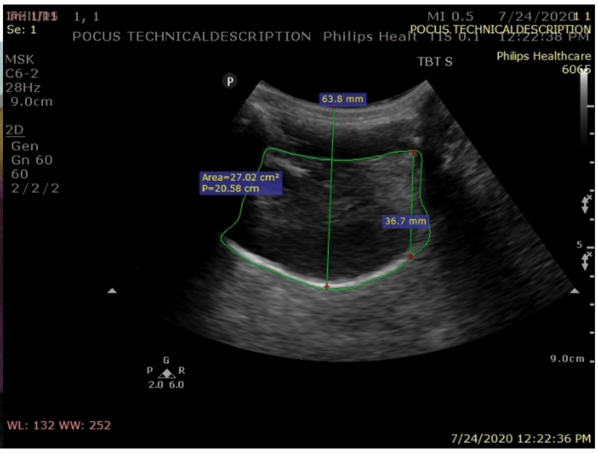

Next, measurements of TBT-S and UAL are completed in the same manner as previously stated while the patient performs the Muller Maneuver.

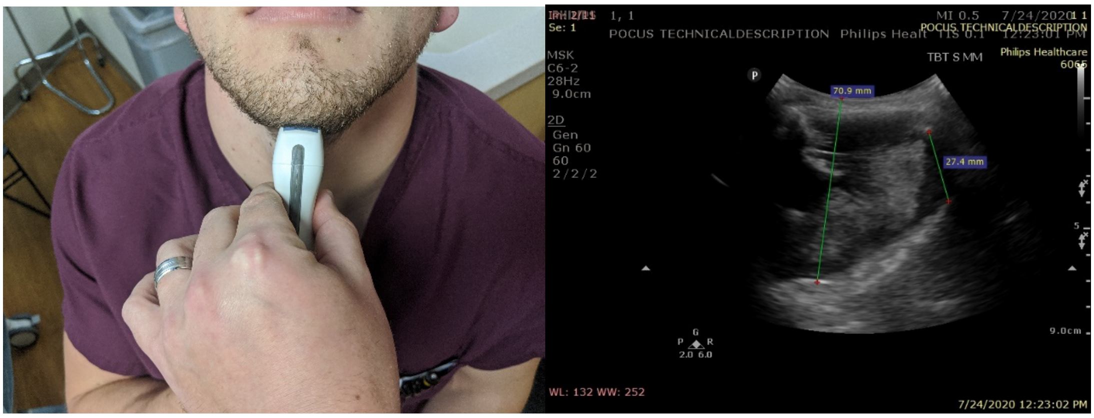

To get the skin-hyoid distance, retroglossal, transverse diameter of the pharynx, and retropalatal views, begin by scout scanning starting in the mental region with the US probe in the coronal plane to ensure the depth settings are appropriate. As you move the probe posteriorly, follow the contour of the submental region and neck which will bring the probe into the transverse plane. Stop once you reach the hyoid bone, make sure you are not applying much pressure and capture an image of the skin-hyoid distance (SHD).

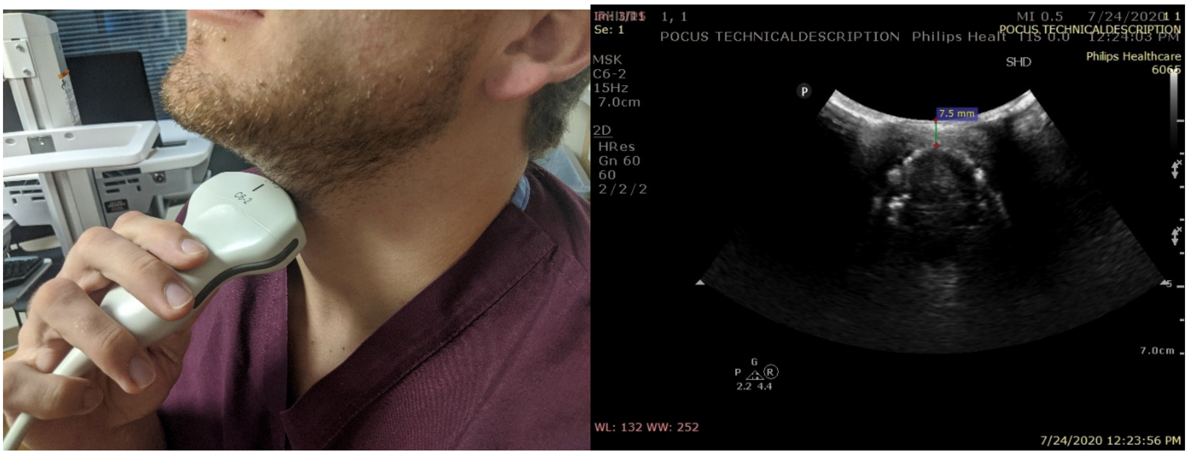

Next, move the probe superiorly to just slip off the hyoid bone, and with the probe in between the coronal and transverse planes image the retroglossal diameter (RGD) - it will be seen as a hyperechoic line.

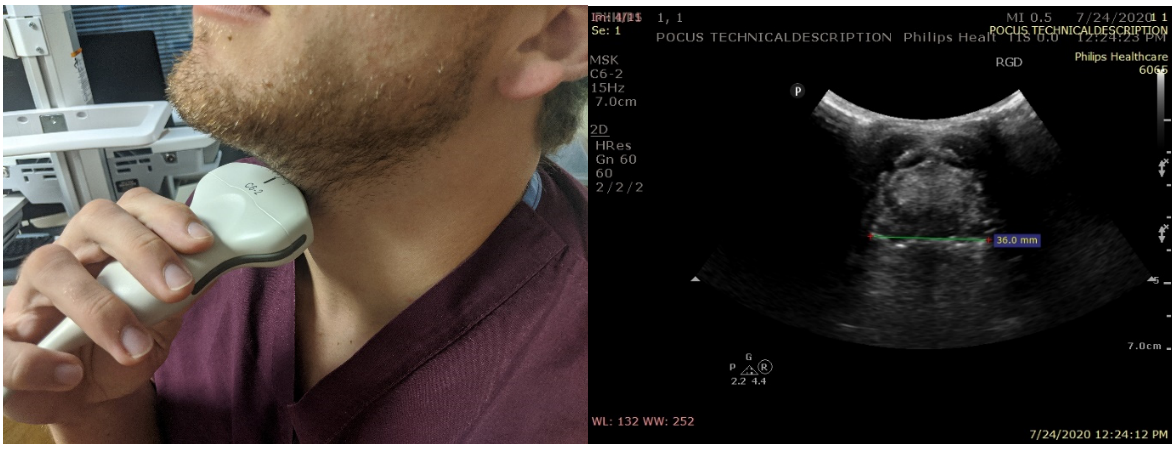

Then, find the hyoid bone again and tilt the probe anteriorly and align it along from the hyoid bone to the external acoustic meatus – this is the transverse diameter of the pharynx (TDP), acquire an image of it.

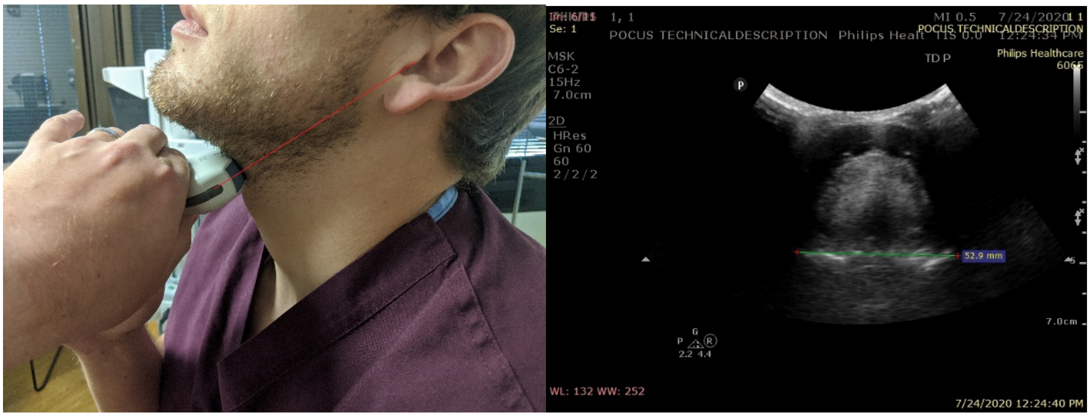

Next, tilt the probe slightly more anterior to visualize the retropalatal diameter (RPD) and acquire an image of it - the soft palate and may also be visible, helping to verify that the hyperechoic line is indeed the retropalatal air column.

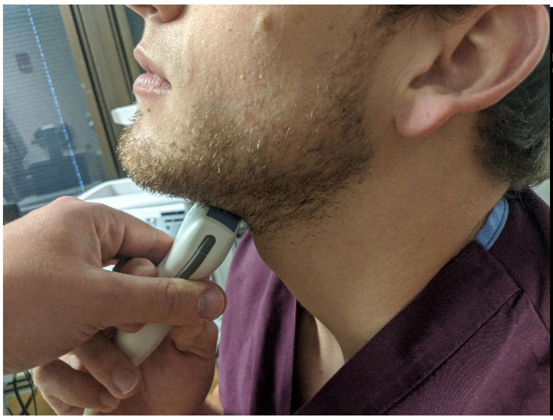

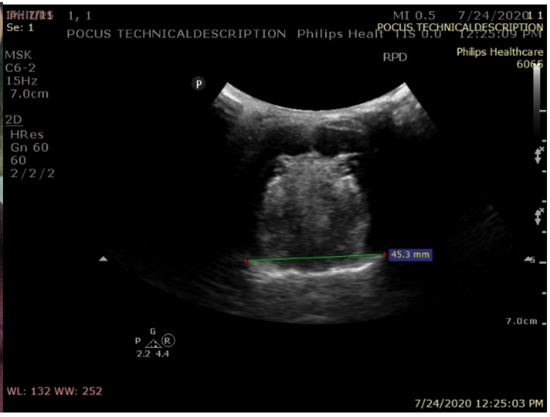

Reposition the probe into the coronal plane within the submental region, adjusting depth to ensure the height of the tongue can be visualized. With the probe coronally oriented, move posterior so that it is just in front of the hyoid bone – acquire an image of the tongue base thickness in the coronal plane (TBT-C). TBT-C is measured from the skin to the maximal thickness of the tongue. Geniohyoid muscle thickness (GMT) can also be measured from the same image, by measuring the thickness of the geniohyoid just lateral to the midline. From here, keep the probe still and try to visualize the lingual arteries. They will be just deep to geniohyoid, near the lateral borders of the tongue. It will likely be necessary to decrease the depth so that pulsations become more visible, and if possible use color doppler to verify their position. Measure the distance between lingual arteries (DLA) from the middle of each artery.

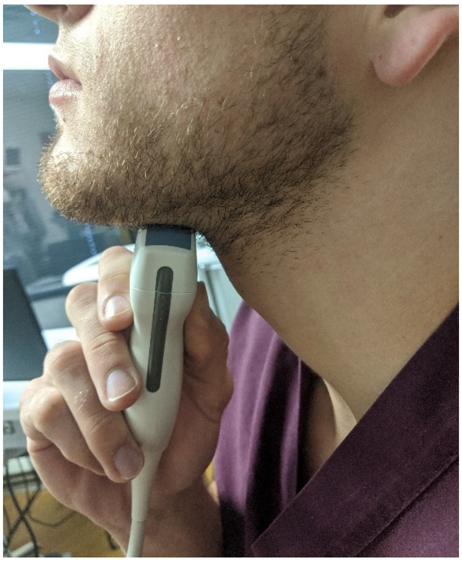

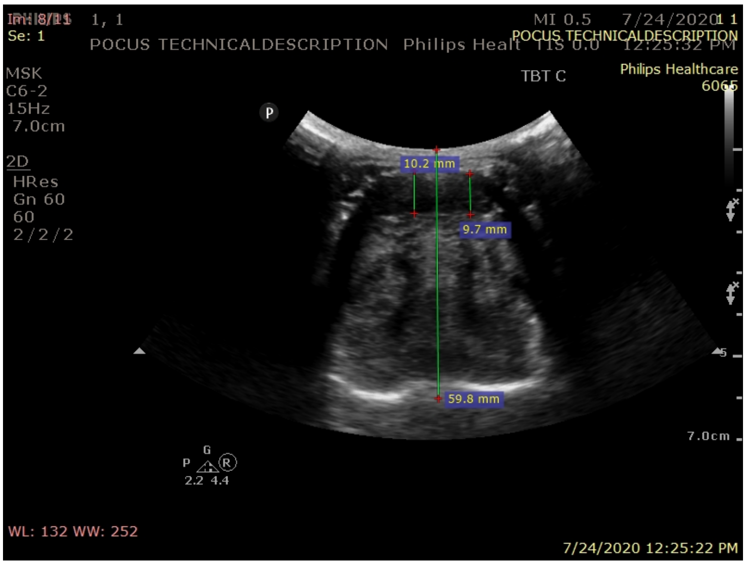

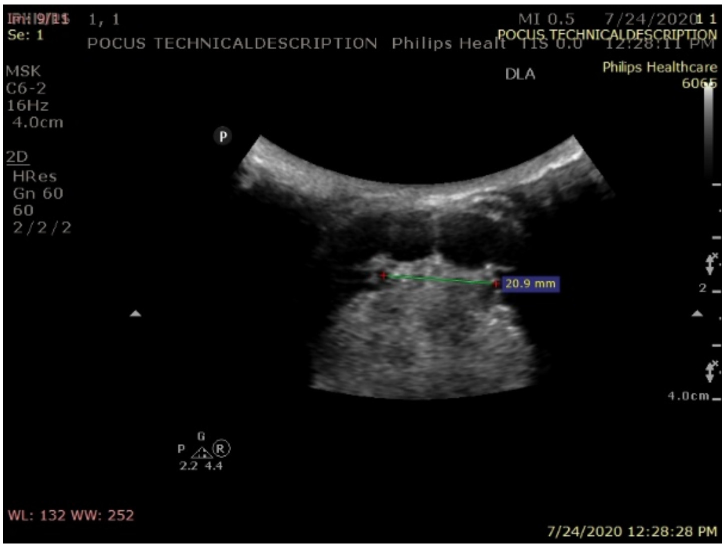

The last 2 images are acquired on the side of the neck and performed the same way on each side respectively. Ask the patient to turn their head away from the side you are scanning. With the probe in the coronal plane, place it on the dorsal/inferior aspect of the mastoid process, posterior of the ramus of the mandible oriented perpendicular to the skin. When the probe is fanned in this view the vertebral bodies should be visible.

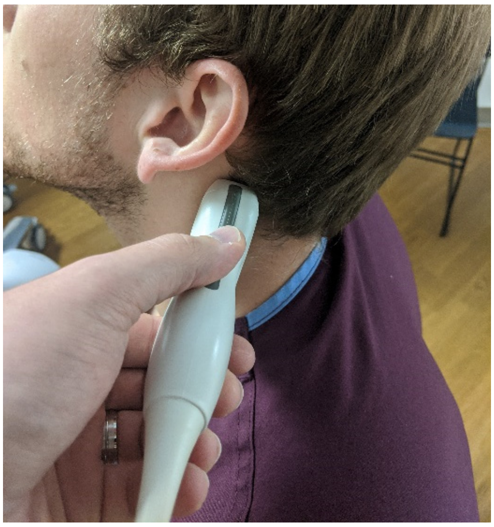

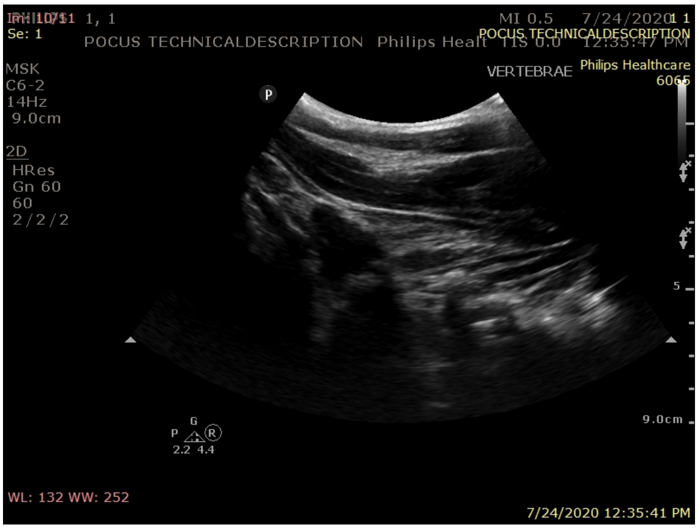

Once the vertebral bodies are viewed, begin to orient the probe anteriorly until the pharynx (hyperechoic white line) is in view. Ask the patient to say their name and visualize the pharynx moving to confirm its location. Then, continue to orient the probe anteriorly until the internal carotid artery (ICA) is in view. Slight fanning of the probe may be necessary once the ICA is viewed to get a more clear image. Slight rotation of the probe may also be necessary to optimize the view of the ICA along its long axis. Ensuring that the probe is at the level of the pharynx, and not too high (nasopharynx) or too low (retropharynx), acquire an image that has both the ICA and pharynx in view – lateral parapharyngeal wall thickness (LPWT). Measure from the hyperechoic line of the pharynx to the nearest edge of the ICA. Repeat on the other side.

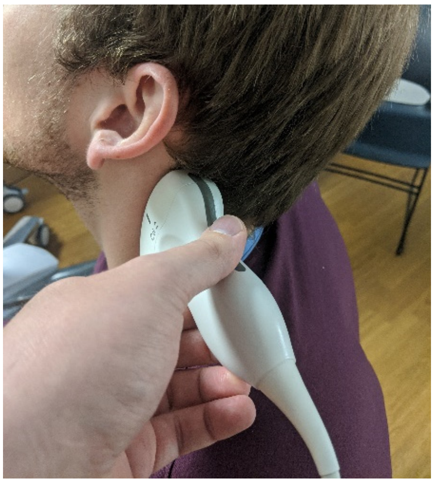 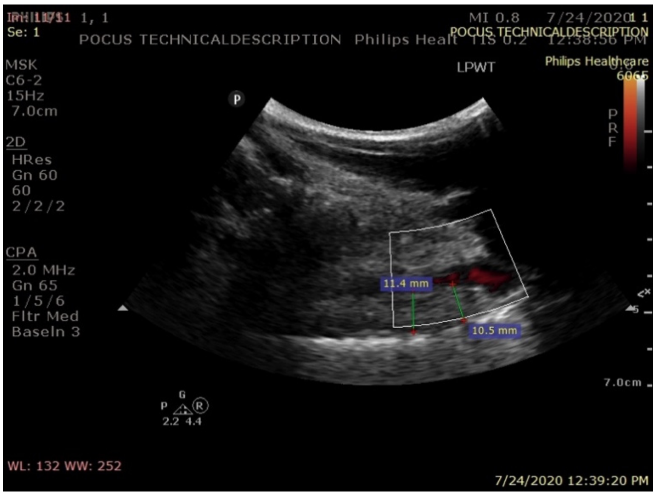

Common Mistakes During POCUS for OSA:

- The subjective nature of US scanning and identifying the landmarks has a learning curve.
- Holding the probe to obtain proper images with the “right” pressure is also important, which takes time to learn, be comfortable with and master over.
- Probe placement and proper alignment can be a barrier to having clear and/or accurate images. E.g. when obtaining the TBT-S, T-CSA, and UAL images (both with and without MM), having the probe in the true mid-sagittal plane is the most difficult part
- These images can also vary greatly based on how the patient positions their tongue, or if they swallow or talk during the process.
- When performing these scans, the patient was often told to be “comfortable”, which will mean different things to each patient. Some had their lips sealed, while others had their lips just slightly open, and their “relaxed” tongue position is certainly different as well.

- Having the patient *properly* perform the Mueller's maneuver (MM) can also be difficult.

- RGD, TDP, and RPD can be difficult as each of these views are dependent on finding the proper angle relative to the hyoid bone. Our RGD, TDP, and RPD scanning protocol was modified from Shu et al.[1]

- The biggest issue with capturing TBT-C is making sure the probe is in a true coronal plane, but just anterior to the hyoid bone. If the image is probe is too far towards the chin then the thickest part of the tongue is not being imaged. And if the probe is not coronal, then GMT and TBT-C measurements are not accurate due to being imaged at an oblique angle.

- Of all the scanning techniques, imaging LPWT is the trickiest image to obtain. Small changes from the position on the side of the neck in the X-, Y-, or Z-plane or the angle of the probe itself can result in suboptimal imaging. Ensuring that the probe position starts posterior to the ramus of the mandible, near the dorsal/inferior aspect of the mastoid process is key. This ensures that the probe is high enough to image the true pharynx. As the probe is tilted anteriorly, the ICA will come into view. The angle at which the probe is held will also change how much of the ICA's long axis is in view. If the probe is positioned too low on the neck, the common carotid may be imaged accidentally. In addition, the internal jugular vein is in the same vicinity as the ICA so using color doppler and/or testing the vessel's compressibility is vital. If there are issues visualizing the

[1] Shu CC, Lee P, Lin JW, Huang CT, Chang YC, Yu CJ, et al. The Use of Sub-Mental Ultrasonography for Identifying Patients with Severe Obstructive Sleep Apnea. PLoS ONE. 2013;8(5):1–7.

airway itself, fanning the probe anteriorly/posteriorly while asking the patient to talk can easily help pickup movement of the air-mucosa interface. In addition, if you believe the airway is in view the CPA setting on the ultrasound machine and asking the patient to take deep breathes will show airflow on color doppler.

Supplementary B:

S1: Supplementary Table S1- Regression Tables Stop BANG, Numerical Data (Negative Binomial Model)

Characteristic	N	MR[1]	95% CI[1]	p-value
tongue base thickness - sagittal (TBT-S) (cm)	123	0.99	0.98, 1.00	0.2
tongue base thickness - sagittal (TBT-S) during Muller Maneuver (cm)	107	1.00	0.99, 1.01	0.8
Tongue base thickness - coronal (TBT-C) (cm)	123	1.00	0.99, 1.01	0.9
Upper airway length (UAL) (cm)	120	1.01	0.94, 1.08	0.8
Upper airway length (UAL) (cm) during Muller Maneuver	101	1.04	0.96, 1.12	0.3
Left lateral pharyngeal wall thickness (L-LPWT) (cm)	120	1.18	0.89, 1.57	0.3
Right lateral pharyngeal wall thickness (R-LPWT) (cm)	119	1.17	0.88, 1.54	0.3
Tongue cross sectional area (T-CSA) (cm2)	121	1.02	1.00, 1.05	**0.020**
Distance between lingual arteries (DLA) (cm)	122	1.12	0.95, 1.32	0.2
Retro-palatal diameter (RPD) (cm)	123	1.07	0.98, 1.18	0.13
Retro-glossal diameter (RGD) (cm)	123	1.02	0.94, 1.11	0.6
Transverse Diameter of the Pharynx	114	1.07	0.98, 1.17	0.13
Geniohyoid Muscle Thickness (GMT) (cm)	117	1.01	0.91, 1.10	0.8
Skin-hyoid distance (SHD) (cm)	123	1.09	0.93, 1.27	0.3

[1]MR = Mean Ratio, CI = Confidence Interval

S2: Supplementary Table S2- Regression Tables Stop BANG, Categorized in to High/Med/Low (Cumulative Logistic Model)

Characteristic	N	OR[1]	95% CI[1]	p-value
tongue base thickness - sagittal (TBT-S) (cm)	123	0.97	0.94, 1.00	**0.028**
tongue base thickness - sagittal (TBT-S) during Muller Maneuver (cm)	107	0.98	0.95, 1.02	0.3
Tongue base thickness - coronal (TBT-C) (cm)	123	1.00	0.96, 1.06	>0.9
Upper airway length (UAL) (cm)	120	1.07	0.78, 1.50	0.7
Upper airway length (UAL) (cm) during Muller Maneuver	101	1.62	1.00, 2.87	0.071
Left lateral pharyngeal wall thickness (L-LPWT) (cm)	120	2.59	0.61, 11.6	0.2
Right lateral pharyngeal wall thickness (R-LPWT) (cm)	119	2.28	0.56, 9.92	0.3
Tongue cross sectional area (T-CSA) (cm2)	121	1.21	1.08, 1.38	**0.002**
Distance between lingual arteries (DLA) (cm)	122	2.55	1.10, 6.27	**0.034**
Retro-palatal diameter (RPD) (cm)	123	1.69	1.05, 2.80	**0.034**
Retro-glossal diameter (RGD) (cm)	123	1.22	0.81, 1.87	0.3
Transverse Diameter of the Pharynx	114	1.29	0.82, 2.06	0.3
Geniohyoid Muscle Thickness (GMT) (cm)	117	6.07	1.23, 38.2	**0.040**
Skin-hyoid distance (SHD) (cm)	123	1.62	0.73, 3.98	0.3

[1]OR = Odds Ratio, CI = Confidence Interval

S3: Supplementary Table S3- Regression Tables AHI (continuous) (Gamma model)

Characteristic	N	MR[1]	95% CI[1]	p-value
tongue base thickness - sagittal (TBT-S) (cm)	74	1.00	0.98, 1.02	0.8
tongue base thickness - sagittal (TBT-S) during Muller Maneuver (cm)	65	1.00	0.99, 1.02	>0.9
Tongue base thickness - coronal (TBT-C) (cm)	74	1.00	0.99, 1.03	0.7
Upper airway length (UAL) (cm)	72	0.93	0.77, 1.13	0.4
Upper airway length (UAL) (cm) during Muller Maneuver	62	1.02	0.85, 1.27	0.8
Left lateral pharyngeal wall thickness (L-LPWT) (cm)	71	1.37	0.60, 3.13	0.4
Right lateral pharyngeal wall thickness (R-LPWT) (cm)	73	1.09	0.51, 2.35	0.8
Tongue cross sectional area (T-CSA) (cm2)	72	1.03	0.98, 1.09	0.3
Distance between lingual arteries (DLA) (cm)	74	0.84	0.53, 1.33	0.5
Retro-palatal diameter (RPD) (cm)	74	1.09	0.83, 1.44	0.5
Retro-glossal diameter (RGD) (cm)	74	1.08	0.86, 1.37	0.5
Transverse Diameter of the Pharynx	68	1.10	0.85, 1.43	0.4
Geniohyoid Muscle Thickness (GMT) (cm)	69	0.90	0.75, 1.26	0.3
Skin-hyoid distance (SHD) (cm)	74	1.05	0.65, 1.74	0.8
Neck circumference (cm)	67	1.05	1.01, 1.09	**0.012**

[1]MR = Mean Ratio, CI = Confidence Interval

S4: Supplementary Table S4- Regression Tables AHI (ordinal) (Cumulative Model)

Characteristic	N	OR[1]	95% CI[1]	p-value
tongue base thickness - sagittal (TBT-S) (cm)	74	0.99	0.96, 1.03	0.6
tongue base thickness - sagittal (TBT-S) during Muller Maneuver (cm)	65	0.99	0.95, 1.03	0.7
Tongue base thickness - coronal (TBT-C) (cm)	74	1.01	0.97, 1.07	0.6
Upper airway length (UAL) (cm)	72	0.99	0.69, 1.42	>0.9
Upper airway length (UAL) (cm) during Muller Maneuver	62	1.25	0.81, 1.95	0.3
Left lateral pharyngeal wall thickness (L-LPWT) (cm)	71	2.75	0.65, 12.0	0.2
Right lateral pharyngeal wall thickness (R-LPWT) (cm)	73	1.81	0.41, 8.01	0.4
Tongue cross sectional area (T-CSA) (cm2)	72	1.08	0.96, 1.21	0.2
Distance between lingual arteries (DLA) (cm)	74	1.00	0.39, 2.55	>0.9
Retro-palatal diameter (RPD) (cm)	74	1.27	0.79, 2.03	0.3
Retro-glossal diameter (RGD) (cm)	74	1.25	0.79, 1.99	0.3
Transverse Diameter of the Pharynx	68	1.53	0.95, 2.52	0.087
Geniohyoid Muscle Thickness (GMT) (cm)	69	0.94	0.64, 1.43	0.7
Skin-hyoid distance (SHD) (cm)	74	1.41	0.60, 3.67	0.5
Neck circumference (cm)	67	1.09	1.01, 1.19	**0.037**

[1]OR = Odds Ratio, CI = Confidence Interval

S5: Supplementary Table S5- Regression Tables - Whether there is AHI Presence (AHI > 5)

Characteristic	N	OR[1]	95% CI[1]	p-value
tongue base thickness - sagittal (TBT-S) (cm)	74	0.99	0.95, 1.04	0.5
tongue base thickness - sagittal (TBT-S) during Muller Maneuver (cm)	65	0.98	0.95, 1.04	0.4
Tongue base thickness - coronal (TBT-C) (cm)	74	1.39	1.00, 3.56	0.4
Upper airway length (UAL) (cm)	72	1.06	0.63, 1.89	0.8
Upper airway length (UAL) (cm) during Muller Maneuver	62	1.33	0.70, 3.01	0.4
Left lateral pharyngeal wall thickness (L-LPWT) (cm)	71	8.89	0.85, 125	0.081
Right lateral pharyngeal wall thickness (R-LPWT) (cm)	73	2.40	0.26, 27.6	0.5
Tongue cross sectional area (T-CSA) (cm2)	72	1.12	0.94, 1.38	0.2
Distance between lingual arteries (DLA) (cm)	74	3.94	0.87, 22.7	0.094
Retro-palatal diameter (RPD) (cm)	74	1.65	0.81, 3.59	0.2
Retro-glossal diameter (RGD) (cm)	74	1.68	0.84, 3.58	0.2
Transverse Diameter of the Pharynx	68	2.47	1.12, 6.32	**0.037**
Geniohyoid Muscle Thickness (GMT) (cm)	69	2.18	0.69, 43.4	0.6
Skin-hyoid distance (SHD) (cm)	74	1.54	0.41, 7.96	0.6
Neck circumference (cm)	67	1.07	0.96, 1.23	0.3

[1]OR = Odds Ratio, CI = Confidence Interval

S6: Supplementary Table S6- Regression Tables - Whether there is Moderate or Severe AHI (AHI > 15)

Characteristic	N	OR[1]	95% CI[1]	p-value
tongue base thickness - sagittal (TBT-S) (cm)	74	0.99	0.94, 1.02	0.5
tongue base thickness - sagittal (TBT-S) during Muller Maneuver (cm)	65	0.99	0.95, 1.03	0.6
Tongue base thickness - coronal (TBT-C) (cm)	74	1.00	0.95, 1.06	>0.9
Upper airway length (UAL) (cm)	72	1.07	0.72, 1.61	0.7
Upper airway length (UAL) (cm) during Muller Maneuver	62	1.30	0.81, 2.22	0.3
Left lateral pharyngeal wall thickness (L-LPWT) (cm)	71	1.56	0.30, 8.41	0.6
Right lateral pharyngeal wall thickness (R-LPWT) (cm)	73	1.42	0.27, 7.64	0.7
Tongue cross sectional area (T-CSA) (cm2)	72	1.05	0.92, 1.20	0.5
Distance between lingual arteries (DLA) (cm)	74	0.84	0.29, 2.39	0.7
Retro-palatal diameter (RPD) (cm)	74	1.22	0.72, 2.12	0.5
Retro-glossal diameter (RGD) (cm)	74	1.12	0.68, 1.87	0.7
Transverse Diameter of the Pharynx	68	1.34	0.79, 2.36	0.3
Geniohyoid Muscle Thickness (GMT) (cm)	69	0.76	0.22, 1.30	0.4
Skin-hyoid distance (SHD) (cm)	74	1.43	0.53, 4.27	0.5
Neck circumference (cm)	67	1.08	0.99, 1.19	0.090

[1]OR = Odds Ratio, CI = Confidence Interval

S7: Supplementary Table S7- Regression Tables - Whether there is Severe AHI (AHI > 30)

Characteristic	N	OR[1]	95% CI[1]	p-value
tongue base thickness - sagittal (TBT-S) (cm)	74	1.00	0.96, 1.04	0.8
tongue base thickness - sagittal (TBT-S) during Muller Maneuver (cm)	65	1.01	0.96, 1.05	0.7
Tongue base thickness - coronal (TBT-C) (cm)	74	1.02	0.96, 1.08	0.4
Upper airway length (UAL) (cm)	72	0.84	0.51, 1.34	0.5
Upper airway length (UAL) (cm) during Muller Maneuver	62	1.13	0.67, 1.86	0.6
Left lateral pharyngeal wall thickness (L-LPWT) (cm)	71	3.75	0.58, 27.9	0.2
Right lateral pharyngeal wall thickness (R-LPWT) (cm)	73	2.16	0.33, 14.5	0.4
Tongue cross sectional area (T-CSA) (cm2)	72	1.08	0.94, 1.26	0.3
Distance between lingual arteries (DLA) (cm)	74	0.58	0.16, 1.91	0.4
Retro-palatal diameter (RPD) (cm)	74	1.15	0.63, 2.15	0.6
Retro-glossal diameter (RGD) (cm)	74	1.18	0.67, 2.12	0.6
Transverse Diameter of the Pharynx	68	1.41	0.78, 2.66	0.3
Geniohyoid Muscle Thickness (GMT) (cm)	69	0.90	0.26, 1.52	0.8
Skin-hyoid distance (SHD) (cm)	74	1.36	0.44, 4.10	0.6
Neck circumference (cm)	67	1.10	1.00, 1.23	0.060

[1]OR = Odds Ratio, CI = Confidence Interval

Supplementary Figure S1- ROC analysis examining sensitivity/specificity of Neck Circumference on Moderate/Severe AHI

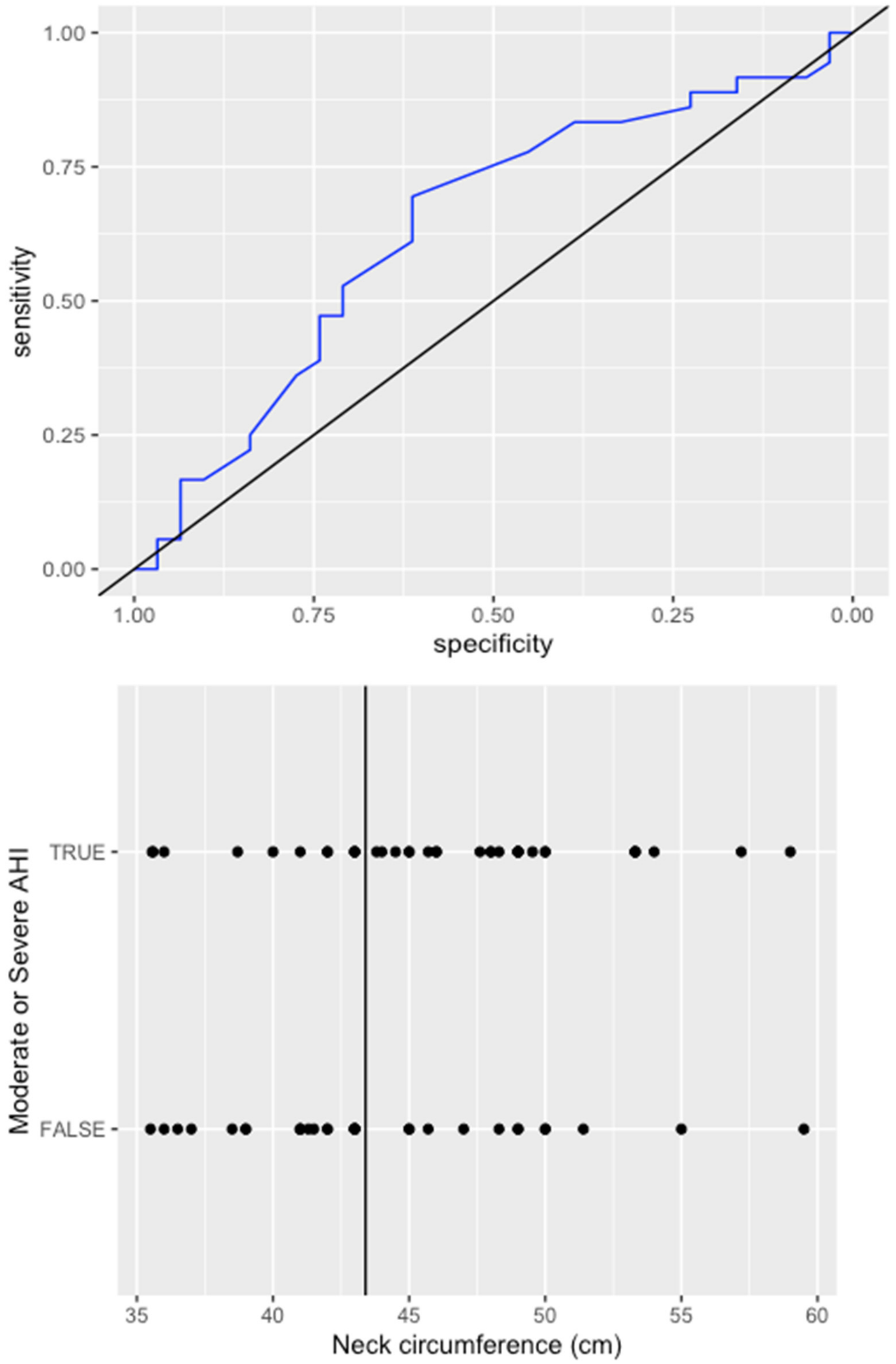

S8: Supplementary Table S8- Relationship between AHI and STOP-BANG (both categorical)

There is a strong relationship between having a STOP-BANG score > 4 and having Moderate/Severe AHI. This comparison has an odds ratio of 6.96, with a Fisher's p-value of 0.0004.

STOP-BANG > 4	Mild or No AHI	Moderate or Severe AHI
FALSE	19	6
TRUE	15	34
